# Training communication abilities in Rett Syndrome through reading and writing

**DOI:** 10.3389/fpsyg.2013.00911

**Published:** 2013-12-06

**Authors:** Rosa Angela Fabio, Ilaria Castelli, Antonella Marchetti, Alessandro Antonietti

**Affiliations:** ^1^Department of Cognitive Science and Education, University of MessinaMessina, Italy; ^2^Research Unit on Theory of Mind, Department of Psychology, Catholic University of the Sacred HeartMilan, Italy; ^3^Service of Learning and Education Psychology, Department of Psychology, Catholic University of the Sacred HeartMilan, Italy

**Keywords:** Rett Syndrome, cognitive rehabilitation, communication, reading, writing, modificability

## Abstract

The goal of this clinical case study is to investigate the possibility of training communication abilities in people with Rett Syndrome (RS). Usually, girls with RS never exceed the sensorimotor stage of development, but the inter-individual variability typical of RS may lead us to doubt the irrevocability of that developmental limit, especially for those girls who are engaged in cognitive rehabilitation. The case study reported here concerns a 21-year-old girl with RS who was engaged in cognitive rehabilitation training based upon the principles of Feuerstein's modificability and mediated learning theory. The training aimed to teach her basic concepts and enhance reading-writing abilities. Statistical analyses showed that the girl reached adequate reading-writing abilities, proving the validity of the cognitive intervention which allowed her to communicate by composing words with her forefinger on an alphabetic table. Although these results need to be cautiously considered as they derive from a single case study, they have implications for future cognitive rehabilitation for deeply impaired clinical conditions as in the case of RS.

## Introduction

Rett Syndrome (RS), named after the Austrian paediatrician who first described it in 1965, is a childhood developmental disorder. Its occurrence is estimated from 1/10,000 to 1/15,000 of newborn females (Kozinetz et al., 1993). It affects mostly females, even though a few cases of males are reported in the literature (Leonard et al., 2001; Cohen et al., 2002).

In the past, RS has been generically associated with the autistic syndrome, but recently it has been recognized as a genetic disorder with a specific biological marker for its diagnosis: its aetiology is due to the genetic mutation of gene MECP2 on the X-chromosome (Amir et al., 1999; Guy et al., 2011). RS is now classified in the category of Pervasive Developmental Disorders (according to the DSM-IV-TR) and there is an open debate about whether to classify RS as also part of the autism spectrum disorder with various efforts to point out the commonalities and differences with autism (Lasalle and Yasui, 2009; Kaufmann et al., 2011; Percy, 2011).

The manifestation of RS is peculiar: the child seems to follow a typical development, but at about 18 months of age a subtle regression in developmental acquisitions begins, opening the path to the clinical stages (Hagberg and Witt-Engerström, 1986). Loss of the previously acquired language skills and of purposeful hand use, increasing difficulties in motor abilities (dyspraxia) and mental retardation are the clearest signs of the regression involved in RS. Other typical signs of RS appear including: hand stereotypies—such as hand-washing, hand-wringing, hand-mouthing—breathing disorders (breath holding and hyperventilation), ataxia, agnosia, bruxism.

RS has gradually become a cross-disciplinary topic of interest as genetic research efforts have mingled with those of psychopathology with the aim of defining the RS phenotype and behavioral condition (Mount et al., 2001, 2002; Berger-Sweeney, 2011; Gadalla et al., 2011; Matsuishi et al., 2011). The high degree of inter-individual variability found among RS patients led Zappella et al. (1998) to propose the “Rett Complex” conception in order to include different degrees of RS impairment while Huppke et al. (2003) described a broad spectrum of phenotypes in RS females from harder forms to milder ones. The problem of RS inter-individual variability is closely related to another well-known problem in RS: the one of assessment. In fact, the impaired clinical picture typical of RS makes it difficult to identify both proper assessment procedures and successful rehabilitation strategies.

With respect to the assessment of RS cognitive functions, a focus on two main areas can be identified in the literature: eye gaze communication (Woodyatt and Ozanne, 1992, 1997; von Tetzchner et al., 1996; von Tetzchner, 1997; Umansky and Watson, 1998; Sandberg et al., 2000; Umansky et al., 2001) and communication by means of a functional analysis of behavior patterns (Roane et al., 2001; Baschina et al., 2002; Cass et al., 2003; Sarimski, 2003; Umansky et al., 2003; Wright et al., 2003; Wales et al., 2004), such as abnormal breathing patterns, hand stereotypies, etc. The overall picture that results is that girls with RS are classified within the third or the fourth stage of sensorimotor intelligence, corresponding, respectively, to a mental age of 4–8 and 8–12 months (Olsson and Rett, 1985, 1990; Olsson, 1987) or exceptionally in the transition to the pre-operatory stage (Lindberg, 1988). Up until now there has not been any work with even a single case study that exceeds this developmental range, as a review by Demeter (2000) about assessment in RS literature confirms. In fact, Demeter (2000) showed that not one of the 34 works about assessment in RS that he considered proved that girls with RS exceed the sensorimotor stage and he himself drew the conclusion that “developing an assessment instrument fulfilling the requirements of a classical test is impossible” (p. 231).

However, studies on cognitive processes, specifically on patterns of attention, seem to contradict such a claim. For example, the analysis of spontaneous non-verbal behaviors indicated as precursors of mentalistic understanding showed that girls with RS had no deficits compared to controls (Antonietti et al., 2005). Moreover, girls with RS display shorter average duration of looking at focal objects and looking away and a longer average length of looking at the caregiver's face as well as at the experimenter's face than the controls (Fabio et al., 2009a,b). Finally, gentle physical containment increases the level of attention to the stimuli of RS patients and decentralizes them from their body (Fabio et al., 2009a,b).

Furthermore, the advent of new technologies has improved the possibilities to study attention and cognitive processes in girls with RS. Baptista et al. (2006) were among the first to use eye-tracking technology in the presentation of two stimuli at a time, a target and a distracter stimulus, asking the child to choose the target stimulus during different cognitive tasks. Girls with RS reported high rates of correct answers, thus suggesting that intentional gaze in girls with RS is measurable and can be used as a path to explore their cognitive performances. Other recent works have focused on the relationship between cognitive and neurophysiological factors showing that age at epilepsy onset and seizure frequency were strongly correlated with neuropsychological outcomes and that age at seizure onset was inversely correlated with the ability to recognize stimuli (Vignoli et al., 2010). Girls with RS were also found to have longer recorded event-related potentials latencies and smaller recorded event-related potentials amplitudes than controls, suggesting slowed information processing and reduced brain activation with advancing years (Stauder et al., 2006). In summary, the overview of cognitive deficit in RS is not yet exhaustive or clear, but girls with RS appear to show intention and preference regarding social and cognitive stimuli, and also seem to have the potential for learning in an intentional way.

With regard to the literature on rehabilitation in RS, behavioral training based on the operant conditioning principles (Lovaas and Leaf, 1981; Smith et al., 1995), as well as intervention in the communication area (Sigafoos et al., 1995; Watson et al., 1996), have been implemented with RS patients. Other interventions have been focused on the role of the environment and of the caregiver (Burford and Trevarthen, 1997; Evans and Meyer, 1999, 2001; Koppenhaver et al., 2001; Tortora, 2001; Ryan et al., 2004; Skoto et al., 2004) and on the use of special programmes and devices (Hetzroni et al., 2002; Lotan et al., 2004), including non-verbal training aimed at teaching basic and complex emotion recognition (Antonietti et al., 2008) in order to understand other people's behavior on the basis of mental states reasoning (Antonietti et al., 2002).

No proposal for proper cognitive interventions is reported, probably because of a general “resignation” that has often characterized works on RS. In fact, it is widely assumed that there is nothing to do with these girls, as “RS girls (…) only acquire a basic level of cognition” (Demeter, 2000, p.231) and “the behaviors that are neurologically driven are not open to modification” (Trevarthen and Burford, 2001, p. 321).

This conclusion will be confuted with the single case study here reported. We want to point out that, if properly trained, RS girls can reach higher developmental levels than those widely accepted in the literature thus far. In order to present our proposal for a new cognitive rehabilitation training, it is necessary to underline the main features of this intervention. The expression “properly trained” refers to precise and well-structured procedures repeated constantly every day for a long time, connected to each other hierarchically so that each level constitutes the ground for the next one. The rehabilitation intervention we describe requires a long term investment, both in resources and in time: the girl has to be submitted to it daily for a long time, working with different caregivers in different contexts (such as teachers or educators at school, parents at home, and so on) in order to transfer what she learned to different persons and situations. Therefore, we shall not describe the communication ability training—which is the focus of this paper—alone: it could not be properly understood and evaluated if it was extrapolated from the global frame of the intervention and from the previous training that has “equipped” the girl for it. Thus, we shall first report on the basic principles of the general intervention, then the training about basic concepts and finally the training about the reading-writing abilities. Finally, we shall show the results of a case study: a girl with RS that has been submitted to this intervention by referring to statistical analyses of the outcomes she has reached in the reading-writing ability training—the one that allowed her to communicate today.

## Case presentation

Francesca (pseudonym) is a 21-year-old young-adult girl with RS. She was born in a non-consanguineous marriage. An uncomplicated pregnancy and a full term normal delivery at a hospital were reported. Regular immunizations were carried out. At birth, her weight and height were normal. Gross motor, fine motor, social and emotional and language milestones were normal during the first 3 years of life. She had normal development up to 20 months when the clinical regression of RS began and she went through the four typical RS stages. The molecular analysis of MECP2 confirmed the presence of the mutation associated with RS (precisely R306C). When she started the intervention, she was 8 years old and she was classified as being in clinical stage IV of RS.

By the age of 8 years, she could walk and run, but she did it for hours while turning inside a room. The baseline examination showed that she could not speak any meaningful words, whereas she used to narrate small sentences earlier on. The mother also reported her lack of attachment with family members, her inability to hold, pick up or grasp things in her hands. She would keep both palms of her hands, one over the other, and would move or rub one hand over the other. Teeth grinding was also reported. She was unable to indicate her need for daily activities such as toilet, passing stools, or for food. Marked cognitive and communicative delays were noted. Features suggestive of severe mental retardation were reported. She had no eye contact and characteristic stereotypied hand movements.

The intervention started at the age of 8 and Francesca followed 4 sessions each lasting 50 min for each week for a period of 3 years. In this first period, she learned pre-training abilities. At the end of the pre-training (at the age of 11) phase, she started the reading and writing program to begin communicating. She followed 4 sessions lasting 50 min for each week for a period of two and a half years (92 weeks plus holiday time) for the discrimination of words and the bi-univocal correspondence between word and image and between image and word. When she was 13 and a half years old she started the training of separation of words into syllables and reconstruction which continued again for 4 sessions lasting 50 min for each week for a period of 1 year (55 weeks plus holiday time). At the age of 14 and a half, she started the training of separating syllables into letters and reconstruction. This training, again for 4 sessions lasting 50 min for each week, lasted 6 months (24 weeks). When she was 15 years old, she started the construction of sentences training and real communication. She is continuing to communicate even now.

## Background

Francesca has reached good levels of autonomy in walking and in everyday activities (eating, dressing and so on) and is also able to communicate non-verbally by reading and composing words with her forefinger on an alphabetic table thanks to cognitive rehabilitation training.

Francesca was submitted to this cognitive rehabilitation intervention thanks to the collaboration among family, school and therapists. Her performances in the most important phases of the reading-writing ability training (reading words, reading syllables, reading letters) are reported in the results section.

Francesca has been treated according to the principles of the Declaration of Helsinki and to the APA ethical standards and informed consent was obtained. The research has been approved by the authors' institutional ethical committee.

### Basic principles of the intervention of modificability and mediated learning

The basic principles which inspired the intervention are the modificability and the mediated learning, both proposed by Feuerstein et al. (1988). They claimed that modificability can be applied despite the presence of severe impairments in the individual or of hard hindrances in his/her living conditions, which are both the case in girls with RS. To help these girls learn, it is not enough to expose them to a rich world of colors, objects of different forms, sounds, and movements without changing their relationship with these stimuli so that they do not remain superficial. In order to change an experience into a source of learning, some components are necessary. These components induce the individual to classify, make comparisons, group, label and convey meaning to the current experience by placing it in relation to previous ones. This active way of experiencing the world is the result of a form of interaction, the so-called “experience of mediated learning.” Mediation means that a change can be caused by another human person (H) that puts him/herself with an active behavior and with precise intentions between the other person (O) and a stimulus (S); therefore, he/she has the role of a mediator. Thanks to an experience of mediated learning, the organism (O) that is directly exposed to the stimuli (S) receives them and answers them with adequate competence only after their features have been selected, framed, modified by an adult human mediator (H). All that the individual will learn is submitted to an order that has been imposed by the adult mediator who determinates the relations among stimuli. In other words, in the educational and rehabilitative relationship the educator should select some stimuli, stress them, convey them in a sequence of time (before and after) and with purpose, put them into a causal and spatial system, convey a special meaning to certain stimuli, propose them many times, cancel other ones, highlight associations among some stimuli and avoid other ones. Finally, it is important that this kind of intervention takes place at three levels: cognitive, emotional, and behavioral. In fact, the ground of this methodology is the human relationship. The relationship is intended both as an instrument of mediation to improve RS girls' abilities and as a way to restore a meaning to the world itself through the interaction with the adult.

In the educational intervention, the relationship with the adult is based upon the principles devised by Feuerstein et al. (1988) and described in Table 1.

**Table 1:** **Basic principles of the intervention of modificability and mediated learning**.

**Basic principle**	**Description**
Unconditioned acceptation	RS girls often have abrupt changes in their arousal, thus influencing their possibility to interact with the external world and, consequently, the way other people interact with them. RS girls perceive the level of acceptation that people have toward them; so, it is very important that caregivers have a disposal of unconditioned acceptance of the girl, to make her feeling loved despite her difficulties in daily interactions.
Rules	Rules are very important keys in this intervention, since they can convey order to the external world, which otherwise would be perceived as chaotic and disorganized-disrupted. Rules can work if they are constantly and repeatedly used in a right way; they must be few and given in an affirmative way (“keep hands still” instead of “do not move your hands”) and must be concrete and given at the right moment.
Reinforcement	Reinforcements are nice events that are able to keep or increase the probability that the behavior they are subsequent to will be shown again. Reinforcements are strictly personal, so it is necessary that educators know what events are nice for each girl: for one girl it can be her favorite snack, for another one her favorite song, and so on.
Containment	RS girls should be bodily-physically contained. For instance their hands have to be kept separate in order to interrupt hand stereotypies and their attention should be driven to the work to be done.
Shaping	Shaping or modeling consists in reinforcing every approximation that is much more similar to the desired behavior, until the girl shows a meta-behavior (a behavior that is close to the desired one) that was not in her behavioral repertoire before.
Fading	Fading consists in giving many helps in the beginning of the work and then in gradually removing them, so that the girl becomes able to do that work without any help.
Neuropsychological area	Selective attention, which is another basic ability that is required by the cognitive training, was enhanced in two steps: the control of body posture and the progressive increase of selective attention's times.

### Training about basic concepts

This training conveys the basis for the reading ability intervention. It is a previous training performed by the girl prior to starting the reading-writing training. Its purpose was to teach the girl various basic concepts in relation to her levels of knowledge in the different areas of the functional diagnosis as described in Table 2.

**Table 2:** **Training about basic concepts**.

**Functional area**	**Goals**
Cognitive area	The girl has learnt the basic discriminations that are necessary to understand reality: she has learnt to recognize common objects, images of common objects, colors, shapes, dimensions.
Emotional and relational area	The girl has learnt to recognize basic emotions and complex ones on her mother's face and then to generalize them on other significant partners' faces (father, teacher, educator).
Linguistic area	After an assessment of the sounds that the girl is able to produce in unintentional way, she was helped to produce them intentionally, for instance related to the expression of a need (e.g., to use the toilet).
Sensorial area	This area covers various sensorial stimulations, such as tactile stimulations of the girl's hands to improve her fine-motor abilities.
Motor-praxical area	Besides precise interventions at the level of great-motor functions (such as physiotherapy, hydrotherapy and horse-therapy) fine-motor functions were taken into account. The most important ability which was trained was eye-hand coordination, which is a basic ability that is required for the cognitive training.
Neuropsychological area	Selective attention, which is another basic ability that is required by the cognitive training, was enhanced in two steps: the control of body posture and the progressive increase of selective attention's times.

### Training about reading-writing abilities

The intervention arose from an initial reflection: if we teach RS girls to communicate through artificial symbols that adults generally do not know—as are those abstract symbols which are usually used with severe multiple disabilities—it would be very hard for these girls to communicate with other adults that do not know that artificial language. In other words, the possibilities for these girls to communicate would remain restricted to very few persons, the ones who share that artificial language. What we conceived by working with RS girls in the cognitive training previously described is that they develop a very good knowledge of pictures, so we wondered: why not try to teach them more abstract symbols, such as our common letters, starting from images in order to allow them to communicate through writing? This was the basic idea that guided the training regarding reading-writing abilities.

The training regarding reading-writing abilities was divided into two main phases: pre-training and training (see Table 3).

**Table 3:** **Training about reading-writing abilities**.

**FIRST PHASE**	
1. Evaluation of pre-requisites	2. Discrimination of images of familiar objects and people
**SECOND PHASE**	
1. Discrimination of the image-word associations	2. Biunivocal correspondence between word and image (direct correspondence) and between image and word (indirect correspondence) See Figure 1
3. Separation of words into syllables and reconstruction See Figure 2	4. Separation of syllables into letters and reconstruction See Figure 3
5. Construction of sentences	6. Communication

#### Pre-training

1. *Evaluation of pre-requisites*. As we have already explained before, Francesca started the reading-writing ability training after being “equipped” with the basic concepts she had learned in the previous training. Moreover, she had learned a way to communicate: she was accustomed to sit down and stay, she was accustomed to the presentation of two stimuli, to the request of looking at them and choosing the requested one either by looking at it and touching it. As far as the pre-requisites of the reading-writing ability training, the most relevant one was the girl's ability to read pictures. Therefore, a group of about 20 images were chosen: they were photos of important persons (parents, siblings, teachers…) and of relevant objects (food, animals, toys…) on an A4 sized sheet of paper.
2. *Discrimination of photos and pictures of familiar persons and objects*. The girl was presented with one photo (target) and a distracter; she was asked to look at them and to choose the target. Stimuli were presented in the randomized right-left order and only when the girl had reached the criteria for each image (she intentionally chose the target, so that the possibility of casual choices could be excluded) the other photos could be introduced with the same procedure. Once this step was reached for all 20 photos, they were reduced to the dimensions of 5 × 5, following the same methods and criteria. Finally photos were substituted by drawings. For instance the photo of the girl's dog was substituted with the drawing of a dog and gradually other images could be introduced, provided that they were part of the girl's world (a chair, a bed, a phone…). The discrimination procedure was the same as for photos.

#### Training

**Figure 1:**
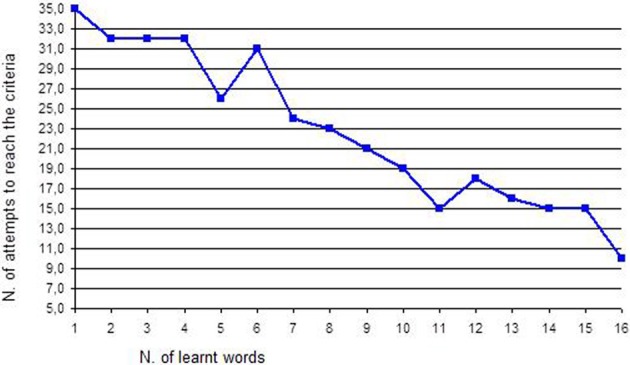
**Number of attempts to reach the criteria for each word**.

**Figure 2:**
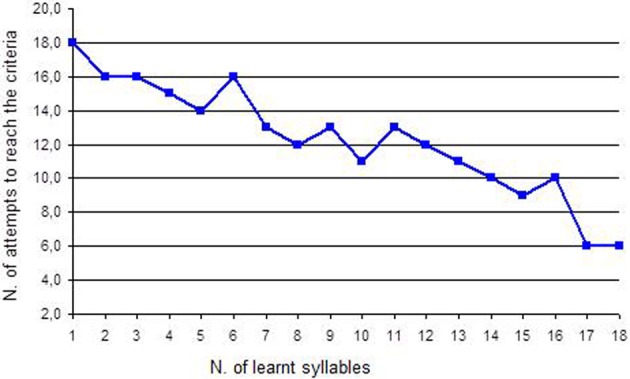
**Number of attempts to reach the criteria for each syllable**.

**Figure 3:**
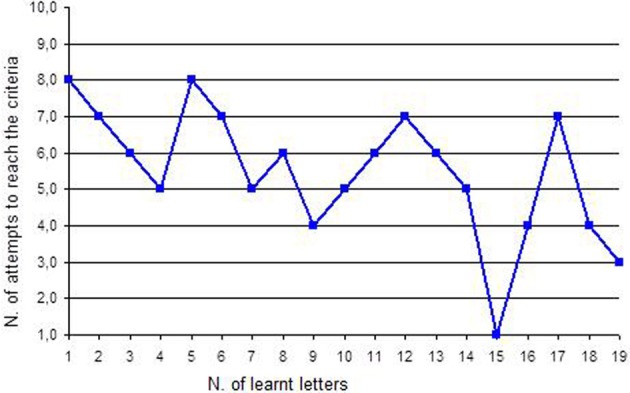
**Number of attempts to reach the criteria for each letter**.

1. *Discrimination of the association image-word*. The work with words started in this phase. Twenty disyllable words were chosen: they were words that interested the girl (such as “dad”) and that were different in the letters (it is better to avoid words that start with “c” and “g” or with “p” and “b” as they are hard to discriminate). Each word was written on a white sheet also reporting, in the first letter, the corresponding image and was presented with a distracter (a white sheet): the girl was asked to choose the stimulus with the word “dad.” The procedure and the criteria were always the same.
2. *Bi-univocal correspondence between word and image and between image and word*. This work provided additional proof of the intentionality of the girl's choices, as it allowed exclusion of casual choices. In the direct correspondence task, the girl was presented with one word, for instance “dad.” and near it the picture of dad and of another object. The girl was asked to choose the right image that corresponded to the word. In the indirect correspondence task, the girl was presented with a picture, for instance “dad.” and near it the words of dad and of other objects (for instance: the word “dad” and another word). The girl was asked to choose the right word that corresponded to the picture. Since the girl's fine motor skills were good, she was also asked to select the right picture and put it on the word and vice versa; otherwise the girl could be helped by the teacher. When the girl recognized the correct picture and word for five consecutive times, the word was learned. The dependent variable was the number of attempts to reach the criteria (5 consecutive correct answers). For example if the first word was “DAD,” as we can see in Figure 1, Francesca tried 35 times before recognizing this word correctly and to associate it with the correct picture.
3. *Separation of words into syllables and reconstruction*. The words that the girl had learned were cut into syllables. The verb “cut” is used here literally: the girl watched the educator cutting words with scissors. For instance “father” was cut into “fa” and “ther”: the girl worked with syllables. She was asked to look at the two syllables (the target syllable “fa” and a distracter syllable) and to choose the target one. In this way, the girl learned the first syllable, then the second, then the two target syllables were presented together and so on for every syllable of each word. After this step, the girl was asked to reconstruct the word: she was given the first syllable (“fa”) and she was asked to choose the following syllable that completed the word “father” choosing between two syllables or even among three. Finally the girl had to make this work without the initial help of the first syllable: she was given some syllables and she was asked to build the word “father.” At the last step, the girl was asked to also build new words that she had not learned in the training but that were made up of the syllables she had learned. The dependent variable was the number of attempts to reach the criteria (5 consecutive correct answers). For example, if the syllable was “DA,” as we can see in Figure 2, Francesca tried 18 times before recognizing it correctly.
4. *Separation of syllables into letters and reconstruction*. The same work with words and syllables was replicated with letters: syllables were cut into single letters. The previously described procedure was also followed with letters and at the end the girl was asked to build new words using letters. The dependent variable was the number of attempts to reach the criteria (5 consecutive correct answers). For example, if the letter was “D,” as we can see in Figure 3, Francesca tried 8 times before recognizing it correctly.
5. *Construction of sentences*. The girl learned to build short sentences using only two words. For instance, she was asked to build the sentence “dad eats” choosing the right letters from a group of many. At the beginning, the girl needed plenty of time, since she had to choose from 21 letters placed in front of her; then she gradually became faster as the position of letters did not change. Sentences gradually became longer.
6. *The quality jump: communication*. Initially the girl was asked simple questions—such as “What is your name?” or “Where is mum?”—and she was encouraged to answer using letters. In this case the quality jump occurred: letters were not used to reproduce what the teacher said anymore, but they were used for the production of an autonomous answer. Finally the single letters were stuck on a big sheet of paper that became the alphabetic table the girl used to communicate. It can be easily rolled up and transported everywhere.

#### Measures

Our procedure was as follows: the girl was sitting at a table and had the target on her right and a distracter on her left. She was asked to look at them and to choose the target by looking at it and touching it (eye-hand coordination). Then the position of the stimuli was changed and the whole procedure was repeated five times with the stimuli in random order (right-left and vice versa). The girl's performance was judged as follows:

- She looks at the target and touches it with her hand: +
- She looks at the distracter and touches it with her hand: −
- She looks at the target (l+ = look+) but touches the distracter (t– = touch-): l+t–
- She is given a little help (i.e., her head is kept still so that she can concentrate her gaze and her hand movement): +.
- She needs considerable help: ±

The scoring system was as follows:

- Positive answers (+, +., l+t–) = 1 point
- Answers that need help (±) = 0.5 point
- Wrong answers (–) = 0 point

We scored l+t– as a positive answer because, given the strong motor impairment in RS, we were aware of the difficulty that these girls have when moving their hand purposefully or to coordinate eye-gaze and hand movement; what was important for us was the correct use of eye pointing as a non-verbal way of communication.

Stimuli were given 5 times in a row in a random right-left order: if the girl answered correctly in each situation, a maximum score of 5 resulted; if all the answers were wrong there was a minimum score of 0. The task was carried out successfully if 5 answers were correct.

Francesca was submitted to the training 3 or 4 sessions a week (lasting 50 min): the rehabilitation training was carried out at school by teachers and educators and at home by the caregiver four times a week. Outcomes were supervised once a week by a cognitive psychologist.

## Results

Performances concerning reading words are described in Figure 1. Francesca learned 16 words: they were names or nicknames of family members (“dad,” “mum,” “efi,” “uncle”), names of familiar objects (“bike,” “ball,” “wine,” “sun,” “moon”), of simple animals (“frog,” “mouse”) and so on (in Italian all these words are disyllable words). To learn the first word (“dad”), Francesca made 35 attempts to reach the criteria, so she needed 7 sessions of work that corresponds to about 2 weeks of training.

In order to control for the statistical significance of the reading words intervention, Test C (Young, 1941), a statistical test for single case research, was used. In this case the obtained value is *Z*_(16)_ = 4.02, *p* < 0.05. A decreasing trend emerged thus confirming that the training did not only enable Francesca to read words, but also allowed her to “learn to learn.” In other words, the girl did not only learn to read some words, but she actually learned reading in general. While the training was going on she was able to learn words faster and sooner.

Performances concerning reading syllables are described in Figure 2. Francesca learned 18 syllables derived from the words she had learned in the previous training. To learn the first syllable (“bi” from “bike”), Francesca made 18 attempts to reach the criteria, so she needed 3 sessions of work that corresponds to about 1 week of training.

Test C produced the value *Z*_(18)_ = 3.72, *p* < 0.05. The trend was decreasing thus confirming again that this training improved both the girl's ability of reading syllables and her ability of “learning to learn.”

Performances concerning reading letters are described in Figure 3. Francesca learned 19 letters. To learn the first letter (“a”), she made 8 attempts to reach the criteria, so she needed 2 sessions of work that corresponds to about 1 week of training.

In Test C, the obtained value was *Z*_(19)_ = 1.94, *p* < 0.05. Also the trend of this intervention was decreasing confirming again the success of this training.

## Discussion and conclusion

Derived from a single case study, these results obviously need to be cautiously considered, but at the same time they have interesting implications for future cognitive rehabilitation for deeply impaired clinical conditions as is the case of RS (Antonietti et al., 2001, 2003, 2004a,b; Rapazzini et al., 2007; Castelli and Marchetti, 2009). First of all, this study supports the possibility to modify RS girls' cognitive structure, improve the quality of their life, as well as the quality of life in the people close to them. If we compare the actual results of Francesca with the single cases reported in the literature, we fail to find the same positive trend. Sometimes this is due to the absence of a follow up, as in the case reported by Bathla et al. (2010), and also in the two cases reported by Malhotra et al. (2002). Sometimes it is because the training was focused on specific cognitive processes such as attention (Fabio et al., 2011).

The positive results we recorded are due to the fact that the training was managed in an effective and comprehensive manner. We are aware of the costs of this intervention in terms of time, energy and patience: it was a long and hard way, full of moments of enthusiasm for every little improvement as well as moments of depression for obstacles and difficulties. But the results we showed here confirmed that it is worth starting such work and continuing along this direction of intervention. The RS girl presented here now composes words using her forefinger as if the table were a computer keyboard; it is important that neither the educator nor the mother touch the girl's hands and arms while she is writing, as she has to do it by herself. She has become very familiar with this kind of communication. We have to say that she generally answers questions, but she hardly ever asks questions. She does so only in those situations that are very important for her and emotionally charged for her, as the following episode can confirm.

She is really affectionate to her little cousin Davide: she wants to stay near him, she follows him, laughs at him and so on. One day, her aunt (Davide's mother) visited Francesca and her family without Davide: as soon as her aunt entered without the boy, Francesca did not greet her, went into her room, took her alphabetic table and asked her aunt “Where Davide?” We need to point out that Francesca had never used the alphabetic table with her aunt before: she must have been really motivated to do so.

There are also other significant episodes in Francesca's life where her parents, her sister, her teachers and educators have been able to understand the reason why she was happy, sad, and angry thanks to this way of communicating that she has learned in her many years of training.

A critical facet of this project is the interaction between school and family, the synergy among parents, teachers, educators, therapists (Antonietti et al., 2009). The success or failure of this intervention largely depends on this critical element: if every one of these persons believes in the possibility of modifying the cognitive level of the girl independently of her impairment, it is possible to work in a well-structured rehabilitation program. As Larsson and Witt-Engerström (2003) already pointed out in their intervention about gross motor ability in RS, expectation, motivation and a joint plan for intervention including all the caregivers of the patient play a role in the success of the work with RS girls. Caregivers themselves play a large part in carrying on training with RS girls: Ryan et al. (2004) showed how important the number and above all the quality of the cues given by the caregivers in order to enhance RS girls' ability to communicate.

Finally, we cannot exclude that the girl's improvements could also be due to other important factors that are independent of the intervention, such as maturational ones or relational ones (for instance, meaningful relations with teachers or educators during the training). In any case, we do not think that these factors alone are enough to explain important results such as the ones reached by Francesca; in fact the difference between a treated girl with RS like Francesca and non-treated ones is very clear and can be logically related to the great deal of time Francesca has spent daily in this intervention that could stimulate her maturational development, or at least could have contained the effects of RS regression.

## Acknowledgements

The publication of this paper has been in part financially supported by AIRETT onlus (Associazione Italiana Rett).

### Conflict of interest statement

The authors declare that the research was conducted in the absence of any commercial or financial relationships that could be construed as a potential conflict of interest.

## Abbreviations

Rett Syndrome: RS.
